# Older Adults and Generational Engagement Across Settings

**DOI:** 10.1093/geroni/igaf122.2995

**Published:** 2025-12-31

**Authors:** Janna Heyman, Henry Davis, Priscilla Allen

**Affiliations:** Fordham University, New York, New York, United States; Fordham University, New York, New York, United States; Louisiana State University, Baton Rouge, Louisiana, United States

## Abstract

The aging population in the United States is steadily increasing and new models of intergenerational and multigenerational engagement are developing in diverse settings. This presentation will discuss innovative generational engagement in community centers, senior centers, assisted living, and nursing homes. Detailed insights obtained through research will be discussed. The presentation will present research from two universities’ ongoing work with community partners. Highlights include key findings from a collaborative study with a large community center providing generational programming and services to older adults, families, and youth throughout New York City. We will introduce ways of bringing generations together in inpatient settings. These approaches will highlight the evolving role and meaning of older adults in generational programming, illustrating their impact on economic mobility, social connections, and overall well-being. Generational programming focused on areas including mentorship, economic empowerment, and co-housing models, emerged as strategies to improve social connections. A qualitative study, using interviews with older adults, families and staff, underscores the importance of autonomy, social connections, financial issues, and inclusion in intergenerational and multigeneration initiatives. The session will include policy and practice implications for social workers and helping professionals to facilitate generational models to support older adults. Best practices including older adults and their lived experiences with community-based initiatives. Participants will gain practical and research-supported insights to generational programming and foster supportive environments.

